# Medical Jurisprudence

**Published:** 1869-04

**Authors:** E. J. Gillett

**Affiliations:** Prof. of Chemistry, Mat. Med. and Toxicology College of Physicians and Surgeons, Keokuk


### MEDICAL JURISPRUDENCE.

### MONOGRAM.

BY E. J. GILLETT, D.D., M.D.,
Prof. of Chemistry, Mat. Med. and Toxicology College of Physicians and Surgeons, Keokuk.

CONSCIOUSNESS is the original source of, and lies back of all belief, and from which there can be no appeal; for there is nothing to which an appeal can be made.

I *am*, or my existence *is*, is known to me by my consciousness; and is capable of no proof, because it is clearer than any proof can make it. That consciousness tells me two facts:

first, that I have a physical organism; and, second, that I think. The first is not material to the question in hand. My thoughts, as effects, are traceable to something that produces them; and that certain something goes by the name of mind. My consciousness informs me that that certain something sees, perceives, analyzes, collocates, arranges, and forms conclusions; also, feels, has joys, sorrows, pleasures, pains—and all these are but developements or manifestations of that one *entity* in action, that one agent—the *I* of my being.

When I compare, it is that one agent that does it; when I judge, or form an opinion, it is an action of the same agent; and if this action relates to what is to be done or not done, it is *will*. When I remember, it is the action of that agent recalling facts which previously have been before the mind. When I have joy or sorrow, it is but the state of that entity in relation to certain things; or, in other words, it is the mind feeling. If this be so, there can be no such thing as dualism, when speaking of mind as mind, as held by Chief Justice Robertson, of Kentucky. To call states of mind a dualism, is a bold absurdity, or, more properly, a solecism. It follows, also, to map off mind, or split it up into attributes, is equally absurd: as those attributes are only the various conditions or states in which the mind is; or, again, in other words, the various directions in which the mind works; or, again, the numberless manifestations of mind. So, the absurdity of a dualism of mind must be patent to all — as much an absurdity as to hold that water, whether congealed or liquid, is not water still. Though it is capable of existing as a gas, vapor, liquid, or solid, it is the product of two elements in equal proportions—hydrogen and oxygen. The elements are the same, whatever the response it may return to the chemist. Its entity is not changed. So in relation to mind. What is usually called the "will," is nothing but the mind deciding. Feeling is nothing but an act of the mind, or, in other words, the mind feeling in relation to a particular thing. Approbation or disapprobation implies intellection; for we cannot approbate unless we perceive, nor perceive, when the question is " to do or not to do," without feeling. Hence, in all cases of intellection on any subject, and feelings consequent, only one agent is concerned; and these are but the manifestations of the one agent — Mind.

So much preliminarily.

The important question which next arises is, What is the basis of responsibility? We answer: It implies power to know what is right, with executive ability. That executive ability is physical or mental efficiency to do. Feelings, or inclinations as named by some, are no party in court, as they have no rights. I have a right to my life, whatever may be the feelings which others have as to my enjoying it. All legislations, human and divine, concur here. For if feelings, and not perceiving, with executive ability, are the rule, then law is a farce; for then law, as generally understood, would cease, and feelings or propensities would become law, which would do away with enactments found in our constitutions as moral beings, or in legislation of aggregates of men. When I am allowed to enter feeling, bias, or disposition as pleas in the defence of wrong doings or acts, I break up all law, and feelings become justifiers for what is done.

Now apply this to the decision of Chief Justice Robertson, in the case of "Smith *vs.* the Commonwealth of Kentucky." (See 1st Duval, p. 230, Ky. Rep.) In that decision the point is made that a disposition or propensity to steal or murder is a legitimate plea for each or both of these acts. The doctrine is this: that so far as a man has a disposition to do a wrong act, that disposition, propensity or feeling, whatever you may call it, is an estopper of judgment, so far as it influenced the act. Now, if on account of this, an abatement may be allowed in little, it may be in much — consequently it may be made to cover all wrong doing.

The corrollary will run thus: A certain degree of wrong will lessen responsibility to the exact amount of or degree of that wrong. The proposition reduced to an algebraic formula will stand thus:

| WRONG. | RIGHT. |
|---|---|
| $\frac{1}{4}$ | $\frac{1}{4}$ |
| $\frac{1}{2}$ | $\frac{1}{2}$ |
| $\frac{3}{4}$ | $\frac{3}{4}$ |
| 1 | 1 |

Wrong becomes right, and right wrong — entire sinfulness is perfect holiness according to the decision of Judge Robertson, Chief Justice of Kentucky.

"*Credat Judæus Appella, non ego.*"

